# TransposonUltimate: software for transposon classification, annotation and detection

**DOI:** 10.1093/nar/gkac136

**Published:** 2022-03-02

**Authors:** Kevin Riehl, Cristian Riccio, Eric A Miska, Martin Hemberg

**Affiliations:** Gurdon Institute, University of Cambridge, Cambridge CB2 1QN, UK; Gurdon Institute, University of Cambridge, Cambridge CB2 1QN, UK; Wellcome Sanger Institute, Wellcome Genome Campus, Hinxton CB10 1SA, UK; Gurdon Institute, University of Cambridge, Cambridge CB2 1QN, UK; Wellcome Sanger Institute, Wellcome Genome Campus, Hinxton CB10 1SA, UK; Department of Genetics, University of Cambridge, Downing Street, Cambridge CB2 3EH, UK; Wellcome Sanger Institute, Wellcome Genome Campus, Hinxton CB10 1SA, UK; Evergrande Center for Immunologic Diseases, Harvard Medical School and Brigham and Women’s Hospital, 75 Francis Street, Boston, MA 02215, USA

## Abstract

Most genomes harbor a large number of transposons, and they play an important role in evolution and gene regulation. They are also of interest to clinicians as they are involved in several diseases, including cancer and neurodegeneration. Although several methods for transposon identification are available, they are often highly specialised towards specific tasks or classes of transposons, and they lack common standards such as a unified taxonomy scheme and output file format. We present TransposonUltimate, a powerful bundle of three modules for transposon classification, annotation, and detection of transposition events. TransposonUltimate comes as a Conda package under the GPL-3.0 licence, is well documented and it is easy to install through https://github.com/DerKevinRiehl/TransposonUltimate. We benchmark the classification module on the large *TransposonDB* covering 891,051 sequences to demonstrate that it outperforms the currently best existing solutions. The annotation and detection modules combine sixteen existing softwares, and we illustrate its use by annotating *Caenorhabditis elegans*, *Rhizophagus irregularis* and *Oryza sativa subs. japonica* genomes. Finally, we use the detection module to discover 29 554 transposition events in the genomes of 20 wild type strains of *C. elegans*. Databases, assemblies, annotations and further findings can be downloaded from (https://doi.org/10.5281/zenodo.5518085).

## INTRODUCTION

**Figure 1.**
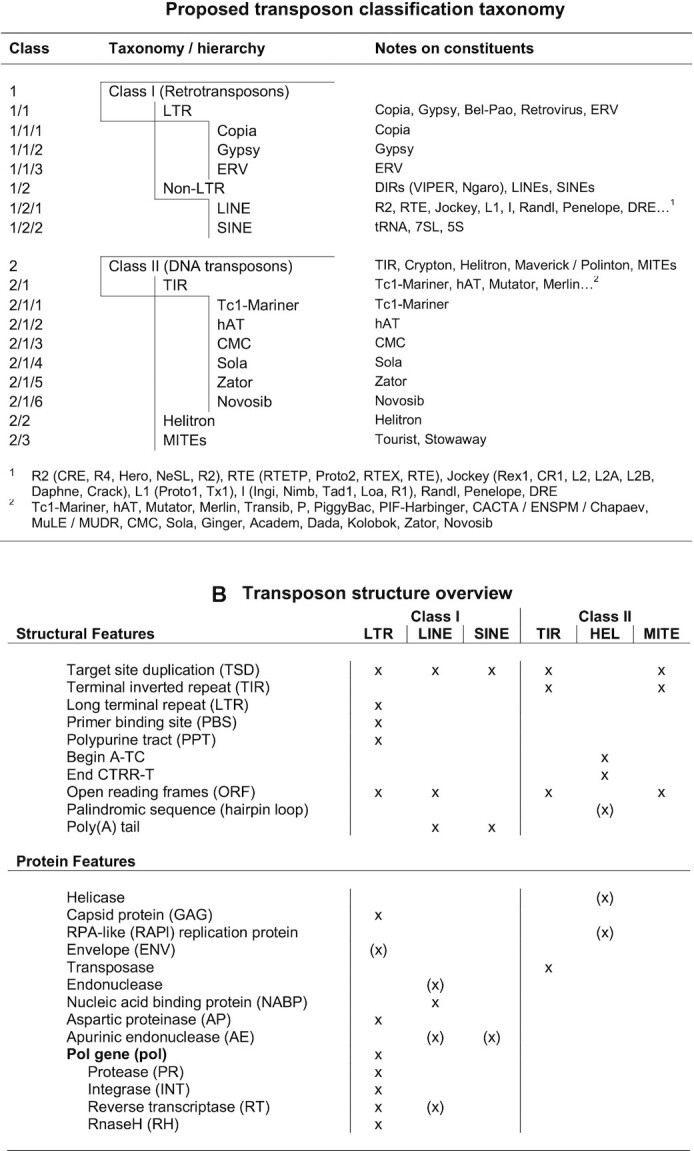
Transposon taxonomy and transposon structure. (**A**) The taxonomy used in this study is based on multiple classification schemes (3,36,49,106) and the taxonomies used by the transposon databases. (**B**) Autonomous, transposition competent transposons have characteristic structural and protein features depending on their class. The proteins are necessary for the transposons to move via class-specific transposition mechanisms. The x mark which structural and protein features are characteristic to different transposon classes and sub classes for complete, autonomous transposons. The (x) mark features that are not required but if present are indicative.

Transposons are evolutionary ancient mobile genetic elements that can move via copy&paste and cut&paste transposition mechanisms. They can be classified within a taxonomic scheme (Figure 1A), and each class is associated with a set of characteristics, e.g. proteins relevant for transposition and structural features (Figure 1B). During transposition, transposable elements (TEs) can leave structural patterns both at the insertion and the deletion site (1–3). Autonomous transposons encode the tools necessary for transposition events, e.g. genes producing transposase, integrase and other enzymes (3), while non-autonomous transposons depend on proteins encoded elsewhere (4). As the insertion of a transposon can be detrimental, many species have developed repression mechanisms, e.g. TE promoter methylation (5) and piRNAs (6). Even though transposition events occur rarely (7), in many organisms large sections of DNA consist of either transposons or their transposition-incompetent descendants that have accumulated mutations over time (4). It is estimated that transposons make up a large share of the genome in many species; 45% in humans, 20% in fruit flies, 40% in mice, 77% in frogs and 85% in maize (8).

Studying TEs is highly relevant for understanding evolutionary processes (9), developmental biology, gene regulation, and many diseases are suspected to be related to transposon activity such as subtypes of haemophilia, immunodeficiency, cancer and Alzheimer’s disease (10–12). Also, TEs are popular for genetic engineering purposes as they allow for direct insertion of their genetic cargo into a target genome (13–15). However, the repetitive nature of transposons and their descendants is a challenge for their analysis and discovery, in particular when using short-read sequencing technologies (7). Long-read technologies facilitate studies of transposons and their functional consequences, but they also require novel computational tools. Although various approaches for identifying transposons have been proposed recently (16), current tools do not provide the flexibility to combine, filter and order annotated elements on a unifying scale, and are often limited to a family of transposons or a group of species (17).

Here, we present a bundle of tools addressing three different tasks related to transposon identification: classification, annotation and detection. The goal of classification is to determine which taxonomic class a given transposon sequence belongs to. The annotation task consists of scanning a genome sequence to mark all transposons. Finally, the detection task involves the comparison of two genomes to identify structural variants arising from the insertion of TEs.

Existing transposon classifiers are difficult to compare directly since they vary in their approach, which features and taxonomies they use, how they evaluate predictions, and which databases are used for training. Applications of SVMs (18), hidden Markov models (19), random forests (20), Gaussian naive Bayes (21), decision trees (22), stacking (23,24), boosting (25,26), neural networks (27–29), evolutionary algorithms (21,30) and genetic algorithms (31–34) can be found in the literature. Most methods use sequence features, such as the k-mer frequency, the occurrence of structural (35) and protein features (18) for classification. Besides, another approach is to classify TEs using the similarity to known transposons based on a sequence library (36).

The annotation of transposons in nucleotide sequences is challenging due to the presence of transposition-incompetent TEs that have been mutated, truncated, degraded, fragmented and dismembered due to nesting (37). Annotation is further complicated by a lack of standards (38) and disagreement on definition, taxonomy and terminology (39,40). Since transposons do not adhere to a universal structure (41), many researchers have employed class-specific approaches (42). Moreover, most of the software employed for transposon annotation was originally designed for gene annotation, neglecting the peculiarities of transposons (39). Existing transposon annotation methods (Table 1) can be assigned to one or more approaches (1,2,41,43). The *de novo* approach finds transposons by identifying repetitive sequences. It is effective in discovering previously unknown transposons with high prevalence (41), but it is computationally costly (39,41), unable to find degraded transposons (41), and risks misidentifying repetitive DNA or high copy number genes as transposons (44,45). The structure-based approach (also called motif-based (42) or signature-based approach (2)) is based on knowledge of the structure of transposons and annotates by finding combinations of characteristic patterns (38,46). This approach enables the discovery of transposition-incompetent transposons thanks to their unique structural properties (41). However, these approaches are often characterized by high false discovery rates (37,44) and they miss transposons with weak signatures (37). The similarity-based approach (also called library-based approach (2)) employs a library of known transposons together with BLAST(-like) tools. The high accuracy (41) and short runtimes (44,47) of this approach come at the cost of its inability to find unrelated transposons (41,47) and the dependency on quality and exhaustiveness of the library (38,44,48). Moreover, the current version of the most widely used database RepBase (49) is behind a paywall and the related tools RepeatMasker and RepeatModeler are not transparent with regards to how transposons were curated and consensus sequences were generated (39).

**Table 1.** Overview of common transposon annotation tools

		Approach			Class I			Class II		
Name		Novo.	Struc.	Simil.	LTR	LINE	SINE	TIR	HEL	MITE
RepeatMasker		x		x	x	x	x	x	x	x
RepeatModeler		x			x	x	x	x	x	x
CLARI_TE	(107)	x	x	x	x	x	x	x	x	x
TESeeker	(41)			x	x	x	x	x	x	x
PILER	(40)	x			x	x	x	x	x	x
Censor	(108)	x			x	x	x	x	x	x
RepLong	(109)	x			x	x	x	x	x	x
EDTA	(44)	x	x	x	x	x	x	x	x	x
MGEScan	(110)	x	x	x	x	x	x			
LTR_Finder	(111)		x		x					
LtrDetector	(112)		x		x					
LTRpred	(73)	x	x	x	x					
LTRharvest	(66)	x	x	x	x					
LTRdigest	(113)		x		x					
SINE-Finder	(68)	x	x				x			
SINE-Scan	(69)	x	x				x			
TIRvish	(67)		x					x		
HelitronScanner	(42)		x						x	
MUSTv2	(70)		x							x
MiteFinderII	(71)		x							x
MITE-Tracker	(72)		x							x
detectMITE	(45)		x							x
MITE-Hunter	(47)		x							x

The most commonly used tools such as RepeatMasker and RepeatModeler cover a variety of transposons, while others focus on certain classes only. The tools use one or more of the *de novo*, structural and similarity-based transposon annotation approaches.

Previous efforts to detect transposition events by comparing two genomes have been based on the analysis of the depth of coverage, discordant and split read pairs (50,51). However, both the task of detecting structural variants (SVs) and annotating TEs are very challenging when using short reads (7). Recently, long-reads technologies have become more widely available, but to the best of our knowledge the only existing method that can take advantage of them for TE detection is LoRTE (52). Although results indicate that LoRTE performs well even on low coverage reads, it is limited to PacBio data and insertion and deletion SVs only.

**Figure 2.**
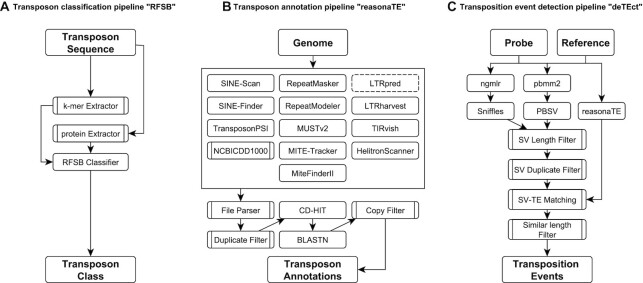
Three pipelines of the TransposonUltimate framework. (**A**) Given the nucleotide sequence of a transposon, relative *k*-mer frequencies (for *k* = 2, 3, 4) and binary protein features are extracted. These features are used by the random forest selective binary classifier (RFSB) to infer the transposon’s class. (**B**) Published transposon and protein annotation tools are applied to a given genome. Resulting annotations are filtered, merged and clustered using CD-HIT. Then, BLASTN is used to find additional full-length copies. (**C**) Sequencing reads obtained using a long-read technology from a probe genome are aligned onto a reference genome using ngmlr and pbmm2. Next, the alignments are used to discover structural variants. After filtering the structural variants, they are matched to the transposon annotations to detect transposition events.

Here, we present TransposonUltimate, a set of tools for the identification of transposons, consisting of three modules for accurate classification, annotation in nucleotide sequences and detection of transposition events (Figure 2). Our new classifier is benchmarked against existing softwares, and we use the annotation module to analyse the genomes of three different species. Finally, the detection module is employed to identify transposition events in 20 high quality genomes from *Caenorhabditis elegans* wild isolates that were assembled using a combination of long- and short-read technologies.

## MATERIALS AND METHODS

### Transposon classification module, RFSB

Given a nucleotide sequence that is considered to be a transposon, the goal is to determine the class of a transposon according to a given taxonomy. This task is a hierarchical classification problem, meaning the classifier needs to identify multiple classes that stand in a relationship described by a taxonomic hierarchy. The design of the classification module includes several aspects; choosing a transposon database for training and testing, feature selection, model structure, training strategy, model implementation, evaluation and benchmarking.

**Figure 3.**
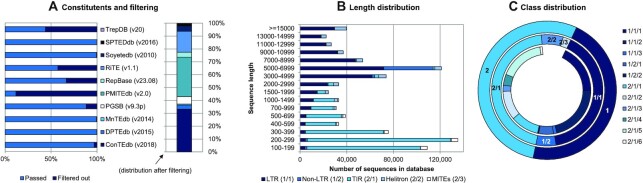
Summary statistics for the TransposonDB. (**A**) Ten publicly available transposon databases were filtered and combined. Sequences with no (valid) class label, fragments, contigs, satellites, RNA, shorter than 100 bp were filtered out. Moreover, duplicates were dropped when merging. Taxonomic schemes by different databases were unified. (**B**) The length distribution of sequences in the databases reveals that most DNA transposons are shorter than 500 bp, while most retrotransposons are longer than 3,000 bp. However, Helitrons are significantly longer than other DNA transposons. (**C**) TransposonDB is balanced in terms of class occurrence, although ERV (1/1/3), SINE (1/2/2) and Novosib (2/1/6) transposons occur rarely.

The classifiers considered here are supervised learning algorithms, and consequently their performance is limited by the data used for training. Previous studies used small transposon sequence databases, each with different taxonomic schemes, which does not allow for a direct comparison. Therefore, we created *TransposonDB* (Figure 3, File F1), a large collection of transposon sequences that consists of ten databases: ConTEdb (53) (http://genedenovoweb.ticp.net:81/conTEdb/index.php), DPTEdb (54) (http://genedenovoweb.ticp.net:81/DPTEdb/browse.php?species=cpa&name=Carica_papaya_L.), mipsREdat-PGSB (55) (https://pgsb.helmholtz-muenchen.de/plant/recat/index.jsp), MnTEdb (56) (http://genedenovoweb.ticp.net:81/MnTEdb1/), PMITEdb (57) (http://pmite.hzau.edu.cn/download_mite/), RepBase (58) (https://www.girinst.org/repbase/, we use version 23.08 that was the last publicly available version before the paywall was introduced), RiTE (59) (https://www.genome.arizona.edu/cgi-bin/rite/index.cgi), Soyetedb (60) (https://www.soybase.org/soytedb/#bulk), SPTEDdb (61) (http://genedenovoweb.ticp.net:81/SPTEdb/browse.php?species=ptr&name=Populus_trichocarpa) and TrepDB (62) (http://botserv2.uzh.ch/kelldata/trep-db/downloadFiles.html). To create the database, the taxonomies were unified, duplicates were dropped and several filter rules were applied (Supplementary Table S1). Filtering included the removal of sequences with no label, the exclusion of fragments, contigs, satellites and RNA sequences. Moreover, only sequences with a length >100 bp and those including at least once each of the letters ‘A’,’C’,’G’ and ‘T’ were kept. To the best of our knowledge, this is the largest database of transposon sequences available. Since TransposonDB covers all relevant Eukaryotic kingdoms, it allows for the training and evaluation of a robust, cross-species hierarchical classification model (Supplementary Tables S2 and S3). Moreover, the database is balanced and covers sufficient examples for all taxonomic nodes (Supplementary Table S4). However, TransposonDB is still likely to be biased as most of the TEs are from plant genomes.

We selected the combination of relative *k*-mer frequencies and binary protein features for our classifier. Relative *k*-mer frequencies represent the number of occurrences of a *k*-mer within a sequence divided by the number of times it would appear if the sequence consisted of this *k*-mer only. Protein features are binary, indicating the presence of a certain protein domain in the sequence. The feature vector consists of *k*-mer frequencies (*k* = 2, 3, 4) and 169 selected domains from NCBI CDD (63) covering class-specific transposons (Supplementary Table S5). We used the 169 domains as query sequences for RPSTBLASTN (v2.10.1) to annotate the conserved domain models at an *e*-value of 5.0 as it performed best in terms of classification performance (Supplementary Figure S1A, B). In addition, two model structures were explored. The binary structure employs binary classifiers for each node (= transposon class) of the taxonomy, and it assigns a probability for each sequence to belong to the node in question. For each internal node, the child is chosen as the node associated with the highest probability. The multilabel structure employs a multilabel classifier for each parent node of the taxonomy with *n*+ 1 classes representing the taxonomic child classes and −1 (return scenario). After inference, the taxonomic class can be determined by choosing the most probable child node at each stage or to return to a higher level and then choose the second most probable child node at that stage. Moreover, we explored two training strategies. The *all* training strategy trains each classification node with the whole training set, while the *selective* training strategy trains each classification child node with a training set that was activated by the parent node. All training strategies, model structures and feature generation were implemented in Python (v3.6.9). Models implementing random forests, AdaBoost, logistic regression, SVM and Naive Bayes from the machine learning package scikit-learn (v0.23) (64) were explored. Random forest consistently yields the highest classification performance (Supplementary Figure S2). Based on these results, we propose a **r**andom **f**orest classifier with a **s**elective training strategy on a **b**inary model structure, *RFSB*.

Previous transposon classification studies use different performance measures, taxonomies, training and testing sets, making it hard to compare them. To evaluate the performance, we consider three perspectives. The first perspective is based on hierarchical precision and recall, meaning it considers the whole taxonomy, as proposed in (65). The second perspective evaluates for different taxonomic levels and the third perspective captures the classification performance of single classes. We benchmark RFSB againts TERL (29), TopDown (24), NLLCPN (27), HC_LGA (33) and HC_GA (31), as their published code allowed for reproduction. To ensure a fair comparison, source codes were partially modified to allow the training and evaluation of these models on the taxonomy used in our work and TransposonDB and can be found on Github https://github.com/DerKevinRiehl/transposon_classifier_rfsb/blob/main/benchmark/ClassifierCode.rar.

### Transposon annotation module, reasonaTE

Given an assembled genome, the goal of the annotation module is to find all transposon occurrences and their locations. Our *reasonaTE* pipeline produces rich annotations, including transposon mask regions (union of all annotated base pairs) as well as transposon annotations, classification, structural and protein features. This is achieved by combining the advantages of thirteen published transposon annotation tools covering different annotation approaches and transposon classes: RepeatMasker v2.0.1 (http://www.repeatmasker.org/), RepeatModeler v4.1.1 (http://www.repeatmasker.org/RepeatModeler/), LTRharvest (66) (https://www.zbh.uni-hamburg.de/forschung/gi/software/ltrharvest.html) and TIRvish (67) (http://genometools.org/tools/gt_tirvish.html) are available as Conda packages. Moreover, we created Conda packages for SINE-Finder (68) (http://www.plantcell.org/content/suppl/2011/08/29/tpc.111.088682.DC1/Supplemental_Data_Set_1-sine_finder.txt), SINE-Scan (69) (https://github.com/maohlzj/SINE_Scan), HelitronScanner (42) (https://sourceforge.net/projects/helitronscanner/files/), MUSTv2 (70) (http://www.healthinformaticslab.org/supp/resources.php), MiteFinderII (71) (https://github.com/jhu99/miteFinder) and MITE-Tracker (72) (https://github.com/INTABiotechMJ/MITE-Tracker) to make them accessible and to facilitate their installation. Also, we include the output files of LTRpred (73) (https://hajkd.github.io/LTRpred/articles/Introduction.html) into the pipeline, as this tool provides high quality annotations, but is available as a Docker image only. As the tools have different output formats, we developed a parser module to convert all outputs to GFF3 format.

After running the annotation tools, additional copies of the identified transposons are searched using the clustering tool CD-HIT (v4.8.1) (74,75) at an identity threshold of 0.9 and BLASTN (v2.10.1) at an e-value of 0.1. If not mentioned further, we used the standard settings for all other parameters of these tools. For the annotation of transposon-characteristic proteins, we have created a Conda packaged version of TransposonPSI (http://transposonpsi.sourceforge.net/), and we also use the protein domains from NCBI CDD for this task. Using TransposonDB, NCBI CDD and RPSTBLASTN, we selected the 1,000 most frequently occurring protein domains that are characteristic to transposons (File F2). As an application, here we annotate the genome *MSU7* of *Oryza sativa* subspecies *japonica* (http://rice.plantbiology.msu.edu/index.shtml), the genome *DAOM197198* of *Rhizophagus irregularis* (https://www.ncbi.nlm.nih.gov/bioproject/?term=PRJDB4945) (76), three reference genomes *VC2010* (https://www.ncbi.nlm.nih.gov/bioproject/?term=PRJEB28388), *N2* (https://www.ncbi.nlm.nih.gov/bioproject/?term=PRJNA13758), *CB4856* (https://www.ncbi.nlm.nih.gov/bioproject/?term=PRJNA275000) and 20 novel wild type strains (77) of *Caenorhabiditis elegans* (Supplementary Table S6).

### Transposition event detection module, deTEct

Given an assembled reference genome and sequenced probe genome reads, the goal is to identify transposition events that are manifested as structural variants. This requires both a list of SVs and annotation of TEs as inputs. We employ the structural variant caller Sniffles on ngmlr (78) alignments and PBSV (https://github.com/PacificBiosciences/pbsv) structural variant caller on pbmm2 alignments of PacBio reads (https://github.com/PacificBiosciences/pbmm2). Moreover, the TE annotations are generated using the proposed reasonaTE pipeline mentioned before.

SVs are filtered twice. First, variants shorter than 50 bp or longer than 1% of the genome length were excluded. Second, duplicate structural variants of the same type are merged. Consecutively, the remaining variants and TE annotations are matched and reported if their length corresponds to each other. Transposon annotations were matched to structural variants if they intersected for at least 10% and their length was similar by a threshold of 50%. We chose to do so as structural variant callers and transposon annotators have an uncertainty regarding exact locations. We therefore consider a similar length more important than a high overlap. The proposed deTEct pipeline is applicable to long-read sequencing technologies, and it has been tested with PacBio data. It has not been tested for short reads and thus we advice against using the pipeline for this type of data.

## RESULTS

### RFSB outperforms other transposon classifiers

**Figure 4.**
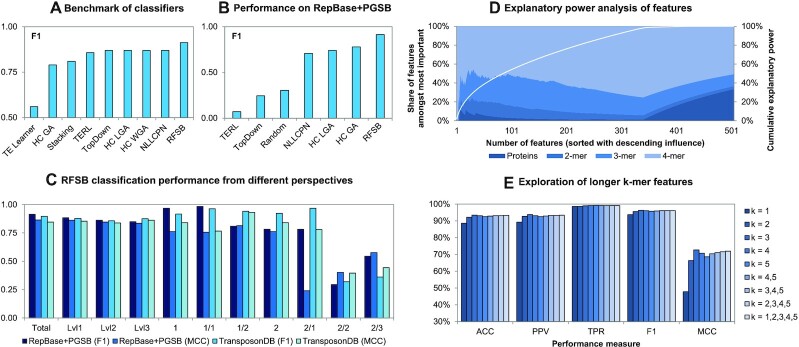
Evaluation of the RFSB classifier. (**A**) Benchmark of different transposon classifiers by reported numbers in publications. (**B**) The performance of selected, reproducable classifiers applied on RepBase+PGSB database using the taxonomy in Figure 1A. Reported numbers represent performances from a total perspective. (**C**) RFSB classification performances from total, taxonomic level and class perspective. (**D**) Analysis of each feature’s contribution to classifier’s explanatory power. The white line shows the cumulative explanatory power. (**E**) Analysis of different *k*-mer features in combination with protein features for a binary classifier differentiating between class 1 and 2 transposons. All values presented were calculated as average across a 10-fold cross validation.

We benchmarked our RFSB method against other transposon classifiers, and the results show that it has the highest sensitivity and specificity (Figure 4A, Supplementary Table S7). TE Learner (20) has the lowest reported performance, while the other methods have similar *F*1 scores. However, this comparison is based on reported numbers from different studies with different evaluation schemes, taxonomies and datasets for training and testing. For a more fair comparison some of the tools were applied to the subset of TranspsonDB which includes RepBase and PGSB (Figure 4B). The comparison of the results reveals large discrepancies. Surprisingly, TERL and TopDown have a performance which is worse than random guessing, and closer inspection of the outputs from NLLCPN reveals that it has learned a constant distribution rather than a relationship between sequences and classes.

A detailed analysis of the classification performance of RFSB across different taxonomic levels and classes reveals a small decrease in performance when considering deeper taxonomic levels (Figure 4C). Underrepresented classes, e.g. Helitrons and MITEs, perform worse, and the results are consistent for both F1 and MCC scores. Moreover, for some classes the performance of RFSB on the large, cross-species TransposonDB is better than for the more homogeneous subset of RepBase and PGSB, which suggests that it is robust, generalisable, and applicable to different species. An inspection of the most informative features (File F3) shows that long *k*-mer features contribute the most to the classification performance, while protein domains have a smaller share amongst the most contributing features (Figure 4D). This motivated the exploration of longer *k*-mer features, but we did not find any significant increase of the performance when using 5-mers (Figure 4E). We also evaluated the runtime (All computations were executed on the cluster CB-GPU1 of the Gurdon institute (OS Ubuntu v18.04.4 LTS). The cluster consists of 80 Intel(R) Xeon(R) Gold 6148 CPUs (2.40 GHz), 315 GB CPU-RAM, two GeForce RTX 2080 GPUs (each 60T RTX-OPS) and 16 GB GPU-RAM.) of the different classifiers, and the results show that the superior classification performance of RFSB comes at a cost of it taking almost twice as long to run as the other methods (Supplementary Tables S10 and S11).

### The ensemble strategy reasonaTE finds more transposons

**Figure 5.**
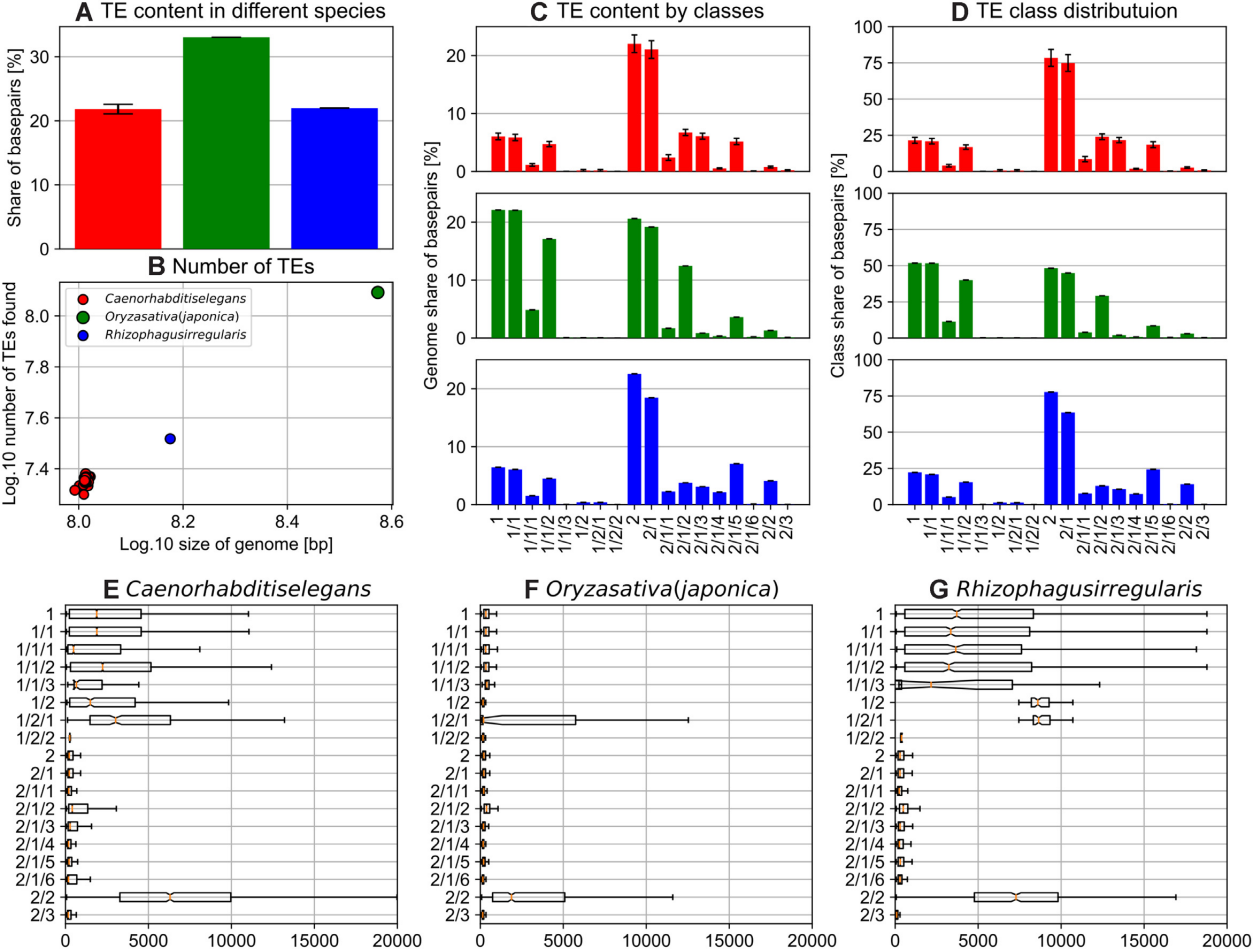
reasonaTE results for three species. The colors used in this figure represent *Caenorhabditis elegans* (red), *Oryza sativa subs. japonica* (green) and *Rhizophagus irregularis* (blue). (**A**) The average TE content of different species. The TE content is calculated as ratio of the sum of all basepairs part of the transposon region mask and the total genome size. The whiskers represent standard deviations. (**B**) The dot size represents the TE content as reported in the first panel, and the figure shows a linear relationship between genome size and the total number of transposons found. (**C**) Average TE content by transposon classes. The values were calculated by dividing the sum of the lengths of all transposons of a specific class by the total genome length. The whiskers represent the standard deviation. (**D**) The class distribution across all TEs based on the number of elements. (E–G) The transposon length distribution by classes for the three species. The boxes cover 25–75% percentiles, including the orange bar at the 50% percentile. The length of whiskers amounts to 150% of the interquartile range.

Next, we evaluated the ability of our reasonaTE pipeline to identify TEs in the genomes of three different species (Figure 5A, B, File F4). The TE content of almost 21% for *C. elegans* is higher than previously reported values of 12% (8), 17% (79) and 12−16% (80). However, as these studies used methods that were biased towards finding specific classes of transposons, it is to be expected that our ensemble strategy finds more TEs. By contrast, the prediction of 33% for *O. sativa*ssp*. japonica* is very close to the mean of other reports (81–89). The content of 23% in *Rhizophagus irregularis* is close to a previous estimate of 27% (90). The low variation of transposon content across different strains becomes obvious for the cluster of *C. elegans*. Interestingly, the relative transposon class frequency reveals clear differences across species (Figure 5C, D). Similarly, the length distributions (Figure 5E−G) exhibit substantial differences between transposons of the same class found in different species. Helitrons in particular vary in length as was observed before (91).

In concordance with (92,93), the share of Helitrons amounts to almost 2% of the *C. elegans* genome. Moreover, the majority of the transposons are TIR DNA transposons, as reported by (79,94,95). Contrary to previous studies (80,96,97), we mainly find hAT, CMC and Novosib transposons to be present in the *C. elegans* genome rather than Tc1-Mariner transposons. Our findings for the rice genome are consistent with previous findings. The high frequency of Gypsy (class 1/1/2) compared to other LTR (class 1/1) and non-LTR (class 1/2) was reported in *Oryza sativa subs. japonica* (87). Moreover, the small share of MITEs, up to 2%, is similar to the previously reported share of 4% (89). A previous study (44) found that class 1 transposons have a larger share (25%) than class 2 transposons (20%) and the frequencies for the subclass level (LTR 23.5% and non-LTR 2%, TIR 17.5% and Helitrons 3.6%) match our findings. Inspection of the annotation density across the chromosomes revealed a characteristic concentration at the arms for *C. elegans* (Supplementary Figure S3), consistent with the higher densities observed for other variants (79,80,98–101).

The comparison of different annotation tools reveals that reasonaTE finds more TEs (Supplementary Figure S4) as none of the other methods finds more than 31.8% of the TEs reported by reasonaTE. In addition, the analysis shows that around 40% of the repetitive elements found by RepeatMasker and RepeatModeler were confirmed as transposons using our approach. Moreover, the transposon characteristic protein annotations by TransposonPSI and the 1000 most frequently occurring proteins from NCBI CDD intersect significantly with reasonaTE’s transposon annotations. The analysis also reveals large overlap between some tools, e.g. MUSTv2 & MITE-Tracker, LTRpred & LTRharvest and SINE-Finder with all other tools.

Closer inspection of the class composition of the TEs found for *Caenorhabditis elegans* confirms the advantages of the ensemble technique of reasonaTE (Supplementary Figure S5). None of the tools is able to find the same share of TEs on its own as the ensemble. Moreover, we find that tools that were designed to identify a specific transposon class annotate TEs from other classes as well.

The runtime of reasonaTE depends on many different factors, including the number of TEs, their distribution, and the size of the genome. In general, the runtime is proportional to the size of the genome, but as we have only examined three different organisms we cannot extrapolate how runtimes will scale. For the investigated genomes in this study, the annotation tasks took around 6 days in the given cluster environment setup. RepeatMasker and RepeatModeler made up the largest share of runtime, the actual post-processing of reasonaTE did not exceed 10% of the total runtime.

### 29 554 transposition event candidates were observed analyzing 20 wild type strains of *Caenorhabditis elegans* using deTEct

**Figure 6.**
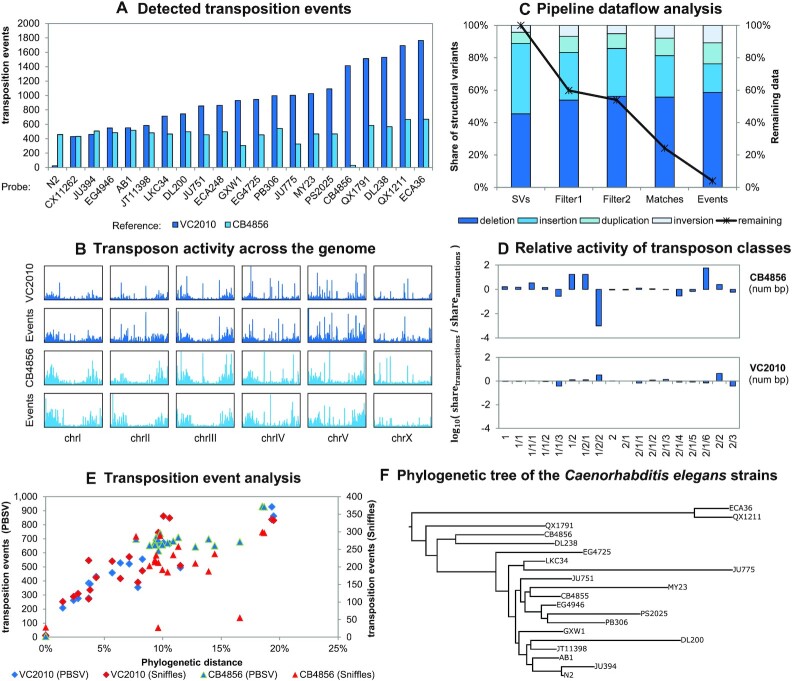
deTEct results and discovered transposition events. (**A**) Results show the number of detected transposition event candidates by probe strain for both reference genomes *VC2010* and *CB4856*. (**B**) The transposon activity in the *Caenorhabditis elegans* genome by chromosomes. The first row shows the density of transposon annotations in *VC2010*. The second row shows the density of transposition events. The following two rows represent results for *CB4856*. For all autosomal chromosomes we identify a characteristic pattern of transposon activity at the ends of chromosomes. (**C**) Dataflow analysis of the pipeline. The diagram shows the share of different structural variant categories at each stage of the pipeline (left y-axis). Deletions make up the largest share of transposition events. Additionally, the share of remaining data is outlined (right y-axis). Approximately 4% of all structural variants initially found are finally identified as transposition events. (**D**) Helitrons and SINEs are more active relative to *VC2010*, while Novosib are especially active relative to *CB4856*. Relative activity is calculated by the share of a class’ basepairs appearing in transposition events divided by its share of a the classes basepairs in the transposon annotation. (**E**) A linear relationship between phylogenetic distance and the number of observed transposition events becomes obvious for the *Caenorhabditis elegans* strains for both SV callers PBSV and Sniffles. Phylogenetic distance is calculated as sum of distances in the phylogenetic tree to the last common ancestor. (**F**) The phylogenetic tree of the *Caenorhabditis elegans* strains. The branch lengths are proportional to the number of polymorphisms that differentiate each pair. Tree based on data from (101).

Finally, we applied the deTEct pipeline to 20 whole genome assemblies of wild type strains of the nematode *C. elegans*. Each strain was compared to the two reference genomes *VC2010* and *CB4856* (Figure 6A, Supplementary Table S8, File F5). As expected, the newly sequenced genomes of these two strains have almost no transposition events when compared to their reference. Closer inspection of the transposon and transposition event densities reveals that the putative transposition events are primarily located at the ends of the chromosomes (Figure 6B) as reported by (79). From the initial list of SVs, 3.97% were identified as transposition events. However, the list included numerous duplicates or very short variants that were subsequently filtered out. Consequently, we find that after filtering, 7.37% of all SVs are caused by transposition events.

Most of the transposition events were observed due to deletions (60%) while insertions, duplications and inversions cause the remaining variation (File F6 + F7). One difficulty in interpreting these proportions stems from the known biases of sequencing data (102) which make insertions hard to detect. This results in an elevated number of observations of cut transpositions (deletions), but fewer paste transpositions (insertions). Nonetheless, we find certain classes of transposons to be especially active in the comparisons of probe and reference genomes, such as Helitrons and SINEs relative to *VC2010*, and LINEs and Novosib when compared to *CB4856* (Figure 6D, File F8). The activity of Helitrons was observed previously (92,93). Helitrons were implicated in the divergence of GPCR genes and heat shock elements. Moreover, they are considered to play an important role in evolution (42). Comparing the two major classes, we conclude that the biggest contribution stems from DNA transposons (82% for VC2010 comparisons and 95% for CB4856 comparisons), similar to the findings in (103).

Moreover, we observe a linear relationship between the number of transposition events found and the phylogenetic distance of the given strains (Figure 6E-F). This result can be observed consistently for PacBio data (Supplementary Table S9). The strains *QX1211* and *ECA36* have the largest differences based on transposon data before (80). Although the identification of SVs and TEs are computationally demanding tasks, the identification of transposition events using deTEct takes only a few seconds to run.

## DISCUSSION

Here, we present TransposonUltimate, a bundle of three modules for transposon classification, annotation and transposition event detection. Moreover, we present TransposonDB, a database containing more than 891 051 transposon sequences from a wide range of species. Our benchmark shows that the classification module RFSB outperforms existing methods. Although *RFSB* has a very high accuracy, we believe that performance could be improved by developing species specific classifiers. It would also be helpful to explore new feature representations that strongly correlate to phylogenetic distance metrics.

The annotation module combines existing annotation approaches using an ensemble strategy, and this ensures a less biased outcome than existing methods that tend to favor certain TE classes. The annotation module could be extended by the search for fragmented copies of annotated transposons connected with filters to avoid false positives. Application to three different species revealed that TEs from the same family vary drastically in length. Thus, an important question for future research is to determine to what extent such differences reflect hitherto uncharacterized families, and to what extent the differences correspond to overall sequence divergence.

The detection module enables the identification of transposition events through structural variants in genomes profiled using long-read sequencing technologies. Application of the *deTEct* pipeline to 20 wild type strains of *C. elegans* suggests that transposon events are responsible for 7.37% of structural variants. Although previous studies have argued that transposons are a major driver of structural variation (102), our results suggest that at least for wild isolates of *Caenorhabditis elegans* this is not the case. As additional high quality assemblies become available, it will be interesting to further explore this important question. Moreover, the development of localisation algorithms of target and donor sites of transposons seems a promising add-on for the detection module. Besides, structural variants gathered from whole genome comparison using anchor filtering (104) could be included and compared.

As long-read technologies are becoming more widely used and the number of sequenced genomes rises quickly, there is an urgent need for methods to identify and annotate TEs which correspond to plurality and in some cases a majority of genome sequences. In particular, as more human (105) and other vertebrate genomes (https://vertebrategenomesproject.org/) are profiled using these technologies, TransposonUltimate will be a valuable tool to improve our understanding of the impact of TEs on both traits and diseases.

## CONCLUSION

Our TransposonUltimate bundle of software tools provides a powerful and user-friendly means of analyzing TEs. In addition to providing highly accurate classifications, our analysis also provides insights as to what features are most informative for predicting TE class. Our ensemble approach to annotation is more unbiased than existing methods that tend to focus on one or a few classes. Finally, our transposition event detection module can take advantage of long-read technologies to identify to what extent TEs underlie SVs.

## DATA AVAILABILITY

Databases, assemblies, annotations and further findings can be downloaded from https://cellgeni.cog.sanger.ac.uk/browser.html?shared=transposonultimate. TransposonDB is available at Zenodo with DOI 10.5281/zenodo.5518085. Source codes, Conda package, installation manual and further documentation and further instructions can be found on https://github.com/DerKevinRiehl/TransposonUltimate.

## Supplementary Material

gkac136_Supplemental_File
