# Supplementary material for: TransposonUltimate: software for transposon classification, annotation and detection
Source: Nucleic Acids Res. 2022 Mar 2;50(11):e64. doi: 10.1093/nar/gkac136 (PMC9226531; doi:10.1093/nar/gkac136)
Supplement: gkac136_Supplemental_File [file gkac136_supplemental_file.pdf]

ADDITIONAL FILES

- **File F1:** TransposonDB.fasta
- **File F2:** NCBICDD1000_Proteins.txt
- **File F3:** Classification_FeatureImportanceAnalysis.csv
- **File F4:** GFF3 files in "PaperSupplements/Annotation/..."
- **File F5:** GFF3 files in "PaperSupplements/Detection/..."
- **File F6:** Detection_SVDistribution.csv
- **File F7:** Detection_PipelineData.csv
- **File F8:** Detection_ClassDistribution.csv

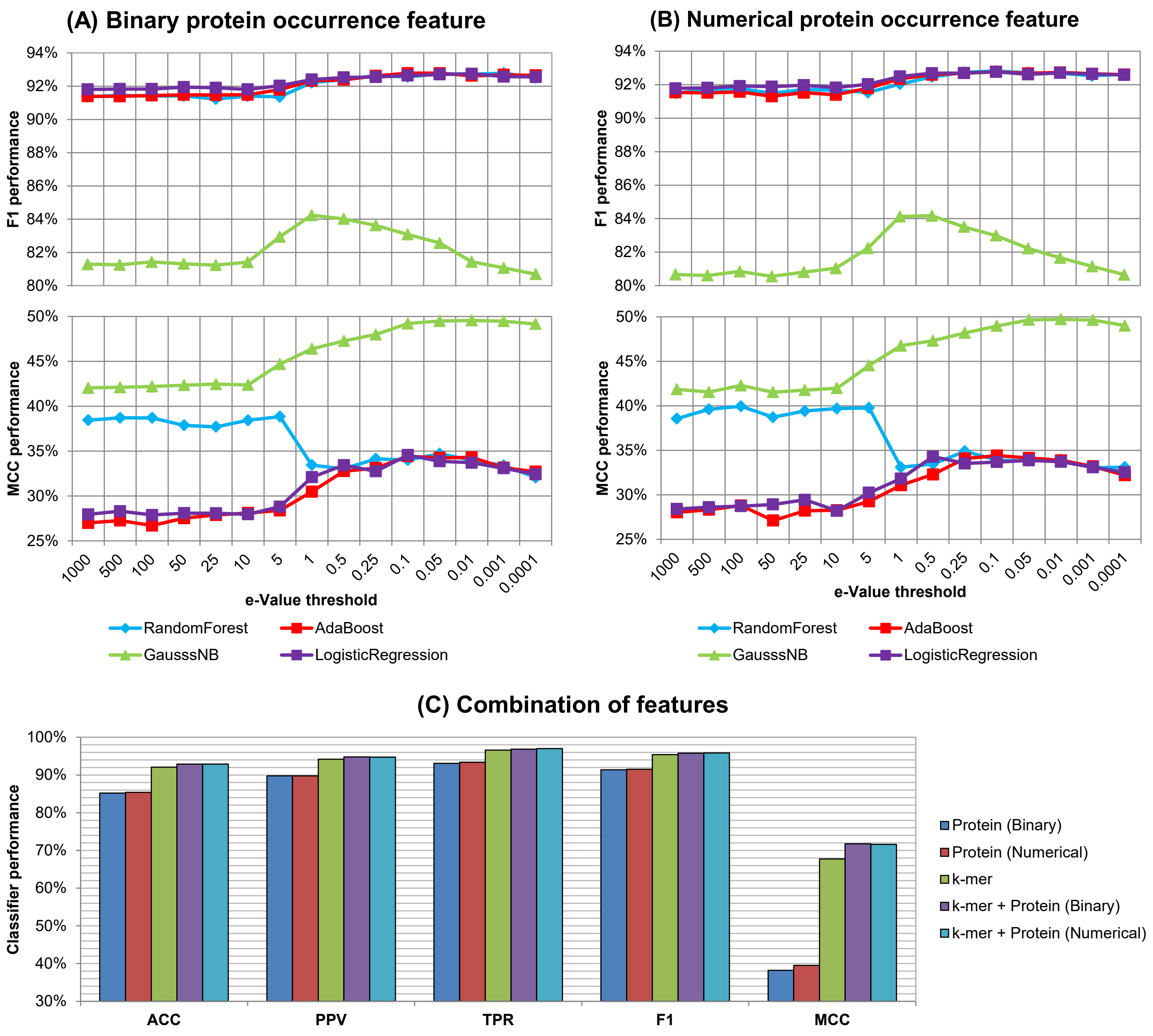

Figure S1. Classification: feature design experiments . All performance measures reported as average across a tenfold cross validation (in %) on RepBase+PGSB dataset for a classifier distinguishing class I and II transposons. (A) Performance of standard classifiers using the binary protein feature for different e-thresholds. This feature is either zero or one depending on whether the protein domain is detected for given e-threshold by RPSTBLASTN. For each experiment, RPSTBLASTN was run with a specific e-threshold. (B) Performance of standard classifiers using the numerical protein features for different e-thresholds. This feature represents the number of times the protein domain is detected for given e-threshold by RPSTBLASTN. (C) Combinations of the protein features with relative k-mer frequency for a random forest classifier.

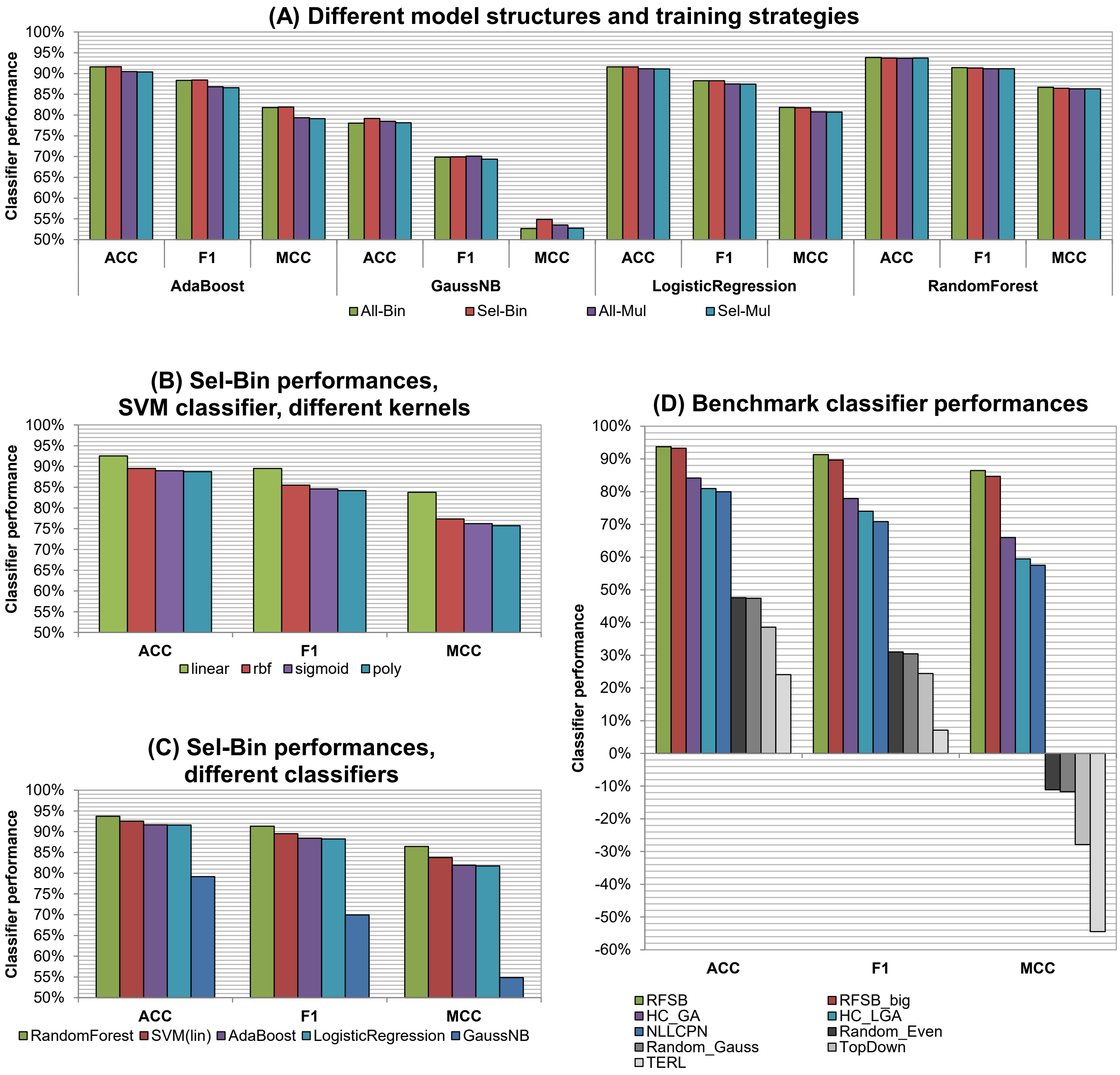

Figure S2. Classification: structure, strategy and model design experiments. All performance measures reported as average across a tenfold cross validation (in %) on RepBase+PGSB from an overall perspective. Panel (A) exhibits the performance of standard classifiers for different model structure and training strategy combinations. Panel (B) shows the performance of SVM classifier model for different kernel functions. Panel (C) summarises the performances of standard classifiers and best SVM classifier for the selective binary (Sel-Bin) combination. Panel (D) compares the proposed ”RFSB” (Random Forest Selective Binary) approach with existing benchmark classifiers. In addition, RFSB trained on TransposonDB is reported as well.

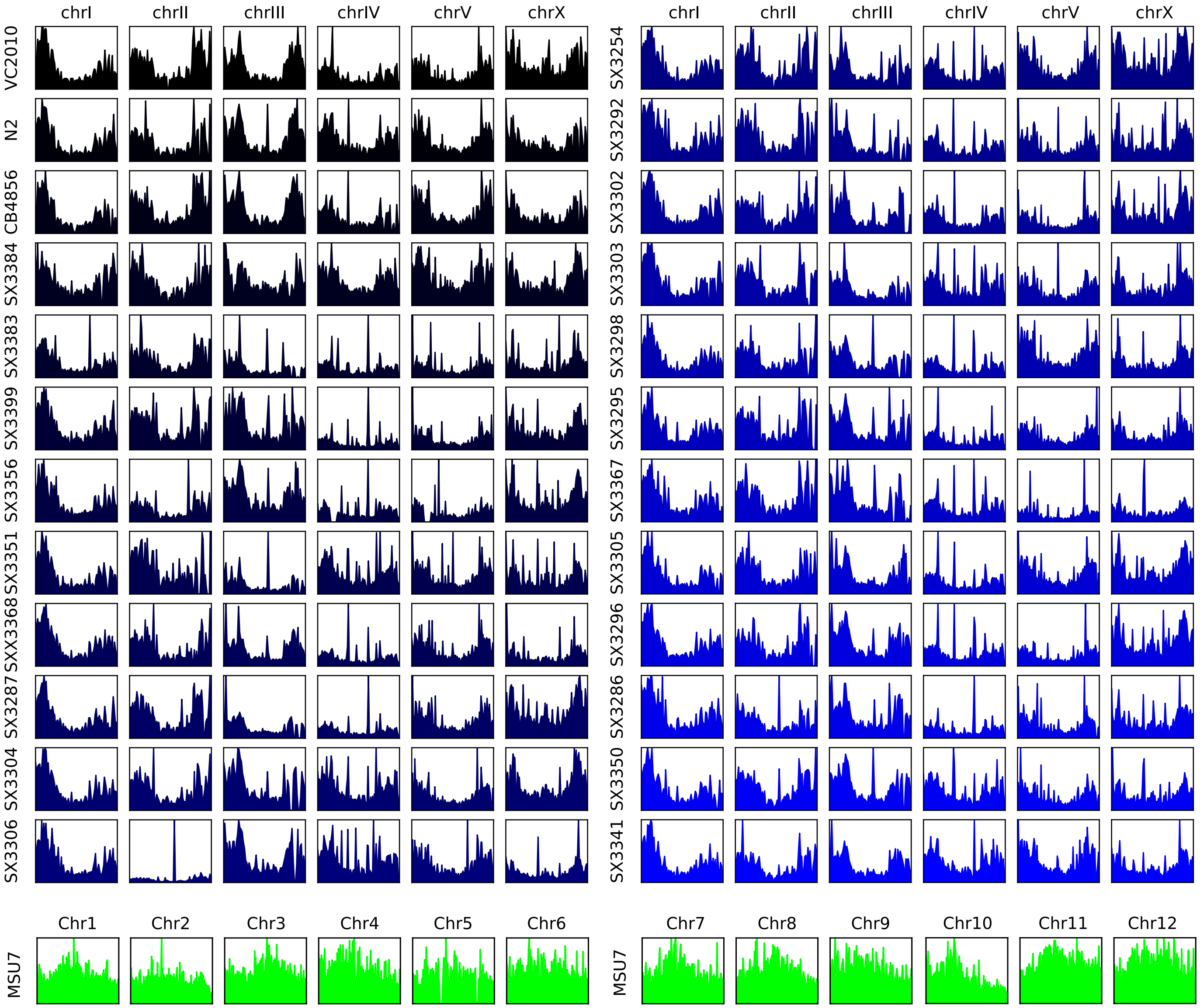

Figure S3. Annotation: transposon annotation density plots. This diagram shows the density of transposon annotations across the chromosomes of the different *Caenorhabditis elegans* and the *Oryza sativa subs. japonica* genomes.

	HelitronScanner	LTRharvest	LTRpred	MiteFinderII	MITE-Tracker	MUSTv2	RepeatMasker	RepeatModeler	SINE-Finder	SINE-Scan	TIRvish	Repeat annotations	Protein annotations	reasonaTE
HelitronScanner	100.00	8.26	2.45	0.88	0.88	6.20	0.25	0.77	0.02	0.41	6.85	7.40	1.69	100.00
LTRharvest	4.96	100.00	13.11	1.99	1.83	7.66	0.57	0.92	0.02	0.44	18.87	8.54	2.48	100.00
LTRpred	4.18	36.82	100.00	1.53	1.32	7.62	0.43	0.95	0.03	0.42	12.98	6.84	3.33	100.00
MiteFinderII	3.15	11.86	3.24	100.00	0.53	6.51	0.11	0.20	0.04	0.47	9.53	5.42	0.04	100.00
MITE-Tracker	4.34	15.02	3.86	0.73	100.00	45.95	0.10	0.21	0.01	0.42	26.92	34.30	0.01	100.00
MUSTv2	5.87	12.22	4.25	1.72	8.90	100.00	0.41	0.74	0.03	0.43	13.01	10.87	1.40	100.00
RepeatMasker	4.10	15.36	4.12	0.49	0.33	6.95	100.00	3.41	0.15	1.77	12.58	12.36	10.07	100.00
RepeatModeler	8.59	17.28	6.20	0.68	0.59	10.24	2.47	100.00	0.01	0.07	19.26	15.33	34.42	100.00
SINE-Finder	68.02	69.08	67.71	67.30	66.74	68.56	67.28	66.72	100.00	89.88	69.10	69.21	66.72	100.00
SINE-Scan	3.73	6.68	2.27	1.20	0.77	4.13	1.00	0.05	3.34	100.00	7.39	5.63	0.85	100.00
TIRvish	4.56	20.84	5.11	1.77	3.63	9.09	0.52	1.11	0.02	0.54	100.00	13.31	1.60	100.00
Repeat annotations	6.05	11.60	3.31	1.24	5.68	9.34	0.63	1.13	0.03	0.50	16.37	100.00	1.07	40.46
Protein annotations	6.45	15.71	7.52	0.04	0.01	5.75	2.38	11.40	0.00	0.35	9.15	4.98	100.00	40.64
reasonaTE	19.15	31.80	11.31	5.35	3.88	20.21	1.19	1.67	0.10	2.09	28.78	9.47	2.04	100.00

Figure S4. Annotation: tool intersection heat map. This heat map presents the intersection of annotations from different tools. The content of cell in row i and column j represents the number of intersecting basepairs of annotations from tools i and j, divided by the total number of basepairs in annotations of tool row i. Numbers are reported in percent, as average across the three reference genomes *VC2010*, *N2* and *CB4856*. Repeat annotations are the combination of annotated repeats by RepeatMasker and RepeatModeler. Protein annotations are the combination of annotated transposon characteristic proteins by NCBICDD1000 and TransposonPSI. The colour of a cell represents its value. Darker colours represent values closer to 100%, while lighter colours represent values closer to 0%.

tool	1	1/1	1/1/1	1/1/2	1/1/3	1/2	1/2/1	1/2/2	2	2/1	2/1/1	2/1/2	2/1/3	2/1/4	2/1/5	2/1/6	2/2	2/3
HelitronScanner	1.06	1.02	0.17	0.85	0.00	0.03	0.03	0.00	2.86	2.60	0.31	0.67	1.39	0.01	0.20	0.01	0.26	0.00
LTRharvest	1.94	1.88	0.49	1.39	0.00	0.06	0.06	0.00	4.94	4.76	0.54	1.75	1.47	0.13	0.84	0.02	0.11	0.07
LTRpred	0.71	0.64	0.10	0.54	0.00	0.07	0.07	0.00	1.74	1.59	0.18	0.65	0.59	0.06	0.11	0.00	0.14	0.00
MiteFinderII	0.00	0.00	0.00	0.00	0.00	0.00	0.00	0.00	1.55	1.55	0.04	0.03	0.17	0.07	1.24	0.00	0.00	0.00
MITE-Tracker	0.00	0.00	0.00	0.00	0.00	0.00	0.00	0.00	0.84	0.84	0.05	0.26	0.13	0.03	0.36	0.00	0.00	0.00
MUSTv2	1.19	1.04	0.10	0.94	0.00	0.15	0.15	0.00	3.25	2.99	0.82	0.68	0.91	0.04	0.55	0.00	0.25	0.01
RepeatMasker	0.03	0.02	0.01	0.02	0.00	0.01	0.00	0.01	0.24	0.23	0.01	0.02	0.11	0.01	0.08	0.00	0.00	0.02
RepeatModeler	0.17	0.15	0.02	0.13	0.00	0.02	0.02	0.00	0.34	0.33	0.07	0.09	0.02	0.01	0.14	0.01	0.00	0.00
SINE-Finder	0.00	0.00	0.00	0.00	0.00	0.00	0.00	0.00	0.03	0.03	0.00	0.01	0.00	0.00	0.02	0.00	0.00	0.00
SINE-Scan	0.03	0.03	0.01	0.02	0.00	0.00	0.00	0.00	0.46	0.46	0.03	0.08	0.03	0.02	0.30	0.00	0.00	0.00
TIRvish	1.18	1.16	0.25	0.91	0.01	0.02	0.02	0.00	4.59	4.49	0.51	2.04	1.10	0.07	0.76	0.02	0.09	0.01
reasonaTE	5.72	5.48	1.00	4.47	0.01	0.24	0.24	0.01	23.13	22.00	2.17	6.73	6.53	0.60	5.89	0.07	0.98	0.16

Figure S5. Annotation: tool class heat map. This heat map presents the share of annotated transposons by different transposon classes. The content of a cell represents the number of basepairs of a specific tool’s annotations related to a specific class divided by the total genomes length. Numbers are reported in percent, as average across the three reference genomes *VC2010*, *N2* and *CB4856*. The colour of a cell represents its value. The darker the colours, the more a tool was able to capture most of the transposons that the ensemble (reasonaTE) found for this specific class.

**database**	**before filtering**	**#F0** no label	**#F1_1** fragments	**#F1_2** contigs	**#F1_3** satellites	**#F1_4** RNA	**#F1** all #F1_*	**#F2** len.100bp	**#F3** alphabet	**#F4** all rules
ConTEdb	322,705	322,702	322,702	322,702	322,702	322,702	322,702	317,109	317,995	312,402
DPTEdb	31,340	31,326	31,326	31,326	31,326	31,326	31,326	31,285	31,325	31,284
PGSB	61,730	61,729	61,729	61,729	61,529	60,813	55,616	60,757	61,397	54,488
MnTEdb	5,925	5,912	5,912	5,912	5,912	5,912	5,912	5,902	5,912	5,902
PMITEdb	2,449,127	357,134	313,211	313,211	313,211	313,211	313,211	298,777	302,529	288,104
RepBase	56,403	53,880	51,674	51,674	51,674	51,648	51,674	51,455	37,796	37,563
RiTE	265,549	264,022	242,216	156,195	241,847	240,509	154,232	152,746	154,058	152,575
Soyetedb	38,664	38,603	38,603	38,603	38,603	38,603	38,603	38,519	38,601	38,517
SPTEDdb	18,413	18,408	18,408	18,408	18,408	18,408	18,408	18,402	18,408	18,402
TrepDB	4,162	3,910	1,874	3,910	3,910	3,910	1,870	3,669	3,869	1,822
TransposonDB										891,051

Table S1. Classification: TransposonDB filter rule application. This table shows the number or remaining sequences in the constituents databases of TransposonDB after the application of the different filter rules.

**databases**	**ConTEdb**	**DPTEdb**	**mipsREdat-PGSB**	**MnTEdb**	**PMITEdb**	**RepBase23.08**	**RiTE**	**Soyetedb**	**SPTEDdb**	**TrepDB**
**Eukaryota**	312,334	29,452	52,691	3,154	282,500	36,681	129,911	36,429	6,733	1,068
**Animalia**	0	0	0	0	0	21,854	0	0	0	20
**Chromista**	0	0	0	0	256	988	0	0	0	74
**Fungi**	0	0	0	0	0	1,961	0	0	0	354
**Plantae**	312,334	29,452	52,691	3,154	282,244	11,721	129,911	36,429	6,733	620
**Protozoa**	0	0	0	0	0	150	0	0	0	0
**UnicellularFlagellate**	0	0	0	0	0	7	0	0	0	0
**Prokaryota**	0	0	0	0	0	54	0	0	0	6
**Virus**	0	0	0	0	0	31	0	0	0	0
**Total**	312,334	29,452	52,691	3,154	282,500	36,766	129,911	36,429	6,733	1,074

Table S2. Classification: TransposonDB sequences across biological domains. This table shows the number of sequences in TransposonDB by the source database and biological domains.

databases	ConTEdb	DPTEdb	mipsREdat-PGSB	MnTEdb	PMITEdb	RepBase23.08	RiTE	Soyetedb	SPTEDdb	TrepDB
**Animalia**	0	0	0	0	0	21,854	0	0	0	20
**Annelida**	0	0	0	0	0	287	0	0	0	0
**Anthropoda**	0	0	0	0	0	8,030	0	0	0	5
**Antropoda**	0	0	0	0	0	1	0	0	0	0
**Ascomycota**	0	0	0	0	0	4	0	0	0	0
**Bilateria**	0	0	0	0	0	1	0	0	0	0
**Brachiopoda**	0	0	0	0	0	1	0	0	0	0
**Chordata**	0	0	0	0	0	10,182	0	0	0	11
**Cnidaria**	0	0	0	0	0	1,228	0	0	0	0
**Ctenophora**	0	0	0	0	0	31	0	0	0	0
**Deuterostomia**	0	0	0	0	0	26	0	0	0	0
**Echinodermata**	0	0	0	0	0	221	0	0	0	0
**Hemichordata**	0	0	0	0	0	37	0	0	0	0
**Mollusca**	0	0	0	0	0	749	0	0	0	0
**Nematoda**	0	0	0	0	0	574	0	0	0	4
**Placozoa**	0	0	0	0	0	8	0	0	0	0
**Platyhelminthes**	0	0	0	0	0	343	0	0	0	0
**Porifera**	0	0	0	0	0	7	0	0	0	0
**Priapulida**	0	0	0	0	0	1	0	0	0	0
**Rotifera**	0	0	0	0	0	123	0	0	0	0
**Chromista**	0	0	0	0	256	988	0	0	0	74
**Ciliophora**	0	0	0	0	0	11	0	0	0	0
**Cliliophora**	0	0	0	0	0	3	0	0	0	0
**Haptophyta**	0	0	0	0	0	15	0	0	0	0
**Myzozoa**	0	0	0	0	0	104	0	0	0	7
**Ochrophyta**	0	0	0	0	0	92	0	0	0	0
**Oomycota**	0	0	0	0	256	760	0	0	0	40
**Orchophyta**	0	0	0	0	0	3	0	0	0	0
**Fungi**	0	0	0	0	0	1,961	0	0	0	354
**Ascomycota**	0	0	0	0	0	749	0	0	0	354
**Basidiomycota**	0	0	0	0	0	1,111	0	0	0	0
**Blastocladiomycota**	0	0	0	0	0	14	0	0	0	0
**Chytridiomycota**	0	0	0	0	0	15	0	0	0	0
**Mucoromyceta**	0	0	0	0	0	9	0	0	0	0
**Mucoromycota**	0	0	0	0	0	71	0	0	0	0
**Plantae**	312,334	29,452	52,691	3,154	282,244	11,721	129,911	36,429	6,733	620
**Angiosperms**	0	29,452	48,583	3,154	257,898	9,997	129,911	36,429	6,733	618
**Bryophyta**	0	0	1,060	0	0	45	0	0	0	2
**Chlorophyta**	0	0	1	0	105	106	0	0	0	0
**Rhodophyta**	0	0	0	0	0	595	0	0	0	0
**Tracheophyta**	312,334	0	3,047	0	24,241	978	0	0	0	0
**Protozoa**	0	0	0	0	0	150	0	0	0	0
**Amoebozoa**	0	0	0	0	0	78	0	0	0	0
**Eozona**	0	0	0	0	0	2	0	0	0	0
**Euglenozoa**	0	0	0	0	0	14	0	0	0	0
**Metamonada**	0	0	0	0	0	42	0	0	0	0
**Percolozoa**	0	0	0	0	0	14	0	0	0	0

Table S3. Classification: TransposonDB sequences across eukaryotic kingdoms. This table shows the number of sequences in TransposonDB by the source database and biological kingdoms.

**database**	**TransposonDB**	**ConTEdb**	**DPTEdb**	**mipsREdat-PGSB**	**MnTEdb**	**PMITEdb**	**RepBase23.08**	**RiTE**	**Soyetedb**	**SPTEDdb**	**TrepDB**
**1**	412,975	252,236	23,832	49,853	1,280	0	25,908	23,508	30,538	4,972	848
**1.1**	360,618	210,831	22,343	48,186	1,267	0	21,542	20,670	30,404	4,769	606
**1.1.1**	122,715	81,329	5,010	11,059	600	0	6,423	4,277	12,482	1,316	219
**1.1.2**	144,221	70,605	9,694	18,497	430	0	9,218	14,490	17,922	3,026	339
**1.1.3**	8,118	4,087	651	0	0	0	3,276	56	0	48	0
**1.2**	51,943	41,405	1,489	1,253	13	0	4,366	2,838	134	203	242
**1.2.1**	47,328	40,679	1,440	931	13	0	3,418	272	134	199	242
**1.2.2**	3,640	0	0	322	0	0	753	2,565	0	0	0
**2**	478,070	60,098	5,620	2,838	1,874	282,500	10,859	106,403	5,891	1,761	226
**2.1**	397,819	131	468	1,416	1,752	282,500	7,791	97,710	5,809	59	183
**2.1.1**	92,563	5	41	261	0	86,195	2,078	2,301	1,645	7	30
**2.1.2**	94,929	5	144	228	135	0	918	93,494	0	5	0
**2.1.3**	19,335	25	108	77	1,084	15,076	2,376	487	65	22	15
**2.1.4**	35,981	0	0	0	0	33,537	0	13	2,370	0	61
**2.1.5**	145,791	4	93	139	285	142,679	755	131	1,664	8	33
**2.1.6**	7,552	0	0	711	0	4,996	467	1,280	65	0	33
**2.2**	56,786	47,199	4,652	14	4	0	665	2,458	82	1,669	43
**2.3**	20,129	12,767	500	475	118	0	2	6,235	0	32	0
**Total**	891,045	312,334	29,452	52,691	3,154	282,500	36,767	129,911	36,429	6,733	1,074

Table S4. Classification: TransposonDB sequences across transposon classes. This table shows the number of sequences in TransposonDB by the source database and transposon classes.

Constituents	NCBI CDD ID
Aspartic proteinase	cd00303, cd05481, cd05484
Apurinic endonuclease	tigr00587
Integrase (core domain)	pfam00665, cog3335, pfam13358, pfam01359
GAG pre-integrase	pfam13976
Ribosomal-processing cycsteine proteinase	cd16332, prk14553
Cysteine proteinase	tigr01586
Peptidase (Prp)	pfam04327
Endonuclease	pfam04231, cog4636, pfam05685, cog2356, pfam01844, pfam05551, pfam07510, pfam13391, pfam13392, pfam13395, pfam14414, prk15137, cd00719, smart00478, cog0648, prk01060, smart00518, prk02308, pfam04493, pfam08459
GAG capsid protein	pfam03732, pfam16297
Helicase	smart00490, smart00487, smart00488, smart00491, cog1201, prk13767, tigr04121, pfam06733, pfam00270, pfam00271, pfam04851, pfam05970, pfam14617, pfam13307
DNA polymerase	cd08637, cd08638, cd08639, cd08640, cd08641, cd08642, cd08643
RNAse H	cd06266, cd09272, cd09273, cd09274, cd09275, cd09276, cd09279
Replication protein A	prk06863, prk06751, prk06752, prk08182, prk06293, prk06461, prk06958, prk07274, prk10053, smart00976, cog0629, cog2965, cog3111, prk05733, cog4085, cog3390, prk06341, prk09010, pfam00436, tigr00621, pfam02765, pfam04057, pfam08646, pfam16686, pfam09104, pfam09103, pfam08661, pfam16900, pfam13742
Reverse transcriptase	pfam13966, pfam07727, pfam00078, pfam11474, tigr04416, pfam13655, pfam17984, pfam17919, pfam17917, pfam13456, pfam06817, pfam06815, cog3344, cd03715, cd03714, cd03487, cd01709, cd01699, cd01651, cd01650, cd01648, cd01647, cd01646, cd01645, cd01644, cd05471
Transposase (incl. DDE domain)	nf033179, pfam13006, pfam14706, pfam02281, pfam13701, pfam13007, pfam13005, pfam04986, pfam03050, pfam01610, pfam01609, pfam01548, pfam01526, pfam18759, pfam18758, pfam17906, pfam13751, pfam13612, cd01187, cd01186, pfam11427, pfam02371, pfam01797, pfam1373, pfam13586, pfam13359, pfam13808, pfam13613
Tyrosine recombinase	cd01196, cd01195, cd01194, cd01192, cd01191, cd01184, cd01188, tigr02224, prk02436, prk00283, cd00796
Others	cd00397, cd00799, cd06094, pfam03564, pfam05380, pfam05585, pfam08284, pfam13975, pfam14223, pfam14244, pfam03184

Table S5. Classification: selection of protein domains This table lists the selected NCBI CDD PSSM model IDs considered for the protein features used in the classification module.

Source	Species	Seq. Technology	#Sequences	Length (BP)	Strain name	Strain location
IRGSP	*Oryza sativa subsp. japo.*	Illumina	12	374,471,240	Nipponbare	Japan
Wormbase WS279	*Caenorhabditis elegans*	Ill., PacBio, Nano.	7	102,092,263	VC2010	Bristol (UK)
Wormbase WS279	*Caenorhabditis elegans*	Sanger	7	100,286,401	N2	Bristol (UK)
Wormbase WS279	*Caenorhabditis elegans*	Illumina	7	98,291,416	CB4856	Hawai (USA)
Cristian Riccio	*Caenorhabditis elegans*	PacBio, Illumina	6	105,149,162	SX3383	Ulupalakua (USA)
Cristian Riccio	*Caenorhabditis elegans*	PacBio, Illumina	6	103,998,030	SX3399	Wuhan (China)
Cristian Riccio	*Caenorhabditis elegans*	PacBio, Illumina	6	104,190,248	SX3356	San Francisco (USA)
Cristian Riccio	*Caenorhabditis elegans*	PacBio, Illumina	6	103,085,866	SX3351	Addisababa (Ethiopia)
Cristian Riccio	*Caenorhabditis elegans*	PacBio, Illumina	6	103,400,028	SX3368	Adelaide (Australia)
Cristian Riccio	*Caenorhabditis elegans*	PacBio, Illumina	6	102,737,178	SX3287	Altadena (USA)
Cristian Riccio	*Caenorhabditis elegans*	PacBio, Illumina	6	102,467,336	SX3304	Amares (Portugal)
Cristian Riccio	*Caenorhabditis elegans*	PacBio, Illumina	6	104,412,447	SX3306	Auckland (New Zealand)
Cristian Riccio	*Caenorhabditis elegans*	PacBio, Illumina	6	102,638,870	SX3254	Bristol (UK)
Cristian Riccio	*Caenorhabditis elegans*	PacBio, Illumina	6	103,222,210	SX3292	Hawai (USA)
Cristian Riccio	*Caenorhabditis elegans*	PacBio, Illumina	6	102,808,004	SX3302	Hermanville (France)
Cristian Riccio	*Caenorhabditis elegans*	PacBio, Illumina	6	102,945,196	SX3303	Lake Forest Park (USA)
Cristian Riccio	*Caenorhabditis elegans*	PacBio, Illumina	6	103,095,877	SX3298	Lisbon (Portugal)
Cristian Riccio	*Caenorhabditis elegans*	PacBio, Illumina	6	102,627,405	SX3295	Madagascar (Madagascar)
Cristian Riccio	*Caenorhabditis elegans*	PacBio, Illumina	6	104,465,563	SX3367	Manuka (USA)
Cristian Riccio	*Caenorhabditis elegans*	PacBio, Illumina	6	103,159,706	SX3305	Palo Alto (USA)
Cristian Riccio	*Caenorhabditis elegans*	PacBio, Illumina	6	102,930,261	SX3296	Roxel (Germany)
Cristian Riccio	*Caenorhabditis elegans*	PacBio, Illumina	6	102,990,752	SX3286	Salt Lake City (USA)
Cristian Riccio	*Caenorhabditis elegans*	PacBio, Illumina	6	103,010,394	SX3350	Southampton (USA)
Cristian Riccio	*Caenorhabditis elegans*	PacBio, Illumina	6	103,231,504	SX3341	Le Perreux-sur-Marne (France)
JST-ACCEL (NIBB)	*Rhizophagus Irregularis*	PacBio	210	149,750,837	DAOM197198	Quebec (Canada)

Table S6. Annotation: case study genomes. This diagram shows source, species, sequencing technology, number of sequences, length in bp, strain name and location for the 25 case study genomes.

(A) Benchmark of classifiers, performances reported in publications of classifiers (different databases and taxonomies)

	Random Even	Random Gaussian	HC_GA	HC_LGA	NLLCPN	TERL	TopDown	RFSB	(TransposonDB) RFSB
**Overall**									
**F1**	31.03 % (0.32%)	30.44% (0.26%)	83.00%	84.00%	90.00%	85.80%	83.00%	**91.34%** (0.33%)	89.66% (0.06%)

(B) Benchmark of classifiers, performances reproduced on RepBase+PGSB database and proposed taxonomy

	Random Even	Random Gaussian	HC_GA	HC_LGA	NLLCPN	TERL	TopDown	RFSB	(TransposonDB) RFSB
**Overall**									
**ACC**	47.58% (0.27%)	47.39% (0.38%)	84.19% (0.41%)	80.94% (0.37%)	79.96% (0.28%)	24.10% (2.91%)	38.60% (23.40%)	**93.75%** (0.24%)	93.28% (0.04%)
**F1**	31.03 % (0.32%)	30.44% (0.26%)	77.91% (0.59%)	74.02% (0.56%)	70.84% (0.38%)	7.11% (0.99%)	24.43% (24.43%)	**91.34%** (0.33%)	89.66% (0.06%)
**MCC**	-11.14% (0.54%)	-11.71% (0.63%)	65.99% (0.86%)	59.45% (0.79%)	57.52% (0.60%)	-54.45% (5.41%)	-27.83% (45.19%)	**86.45%** (0.52%)	84.68% (0.09%)
**Level 1**									
**ACC**	79.23% (0.09%)	79.52% (0.07%)	92.86% (0.13%)	91.78% (0.27%)	92.32% (0.10%)	82.18% (0.54%)	77.99% (5.75%)	**96.48%** (0.10%)	96.11% (0.02%)
**F1**	29.52% (0.3%)	29.14% (0.23%)	75.05% (0.47%)	71.17% (0.51%)	70.19% (0.39%)	0.10% (0.09%)	23.05% (21.99%)	**88.30%** (0.32%)	87.74% (0.07%)
**MCC**	17.35% (0.36%)	17.18% (0.26%)	70.92% (0.53%)	66.44% (0.67%)	66.70% (0.45%)	-7.36% (0.64%)	10.31% (25.19%)	**86.27%** (0.38%)	85.44% (0.09%)
**Level 2**									
**ACC**	82.60% (0.09%)	82.91% (0.05%)	93.63% (0.13%)	92.72% (0.26%)	93.26% (0.08%)	89.21% (0.09%)	82.33% (3.92%)	**96.99%** (0.09%)	96.67% (0.02%)
**F1**	17.57% (0.38%)	17.13% (0.23%)	68.51% (0.73%)	63.70% (0.70%)	60.18% (0.43%)	00.00% (0.00%)	12.89% (20.23%)	**86.30%** (0.40%)	85.66% (0.08%)
**MCC**	7.85% (0.43%)	7.62% (0.24%)	65.12% (0.75%)	59.89% (0.78%)	59.21% (0.48%)	-1.01% (0.50%)	3.21% (22.08%)	**84.65%** (0.45%)	83.81% (0.09%)
**Level 3**									
**ACC**	86.30% (0.05%)	86.57% (0.04%)	92.87% (0.24%)	91.69% (0.22%)	90.91% (0.00%)	90.90% (0.01%)	86.96% (3.16%)	**97.33%** (0.11%)	97.76% (0.02%)
**F1**	9.63% (0.41%)	9.32% (0.34%)	49.93% (2.77%)	38.67% (4.62%)	0.00% (0.0%)	0.00% (0.00%)	7.96% (12.97%)	**84.95%** (0.59%)	87.51% (0.10%)
**MCC**	2.59% (0.42%)	2.56% (0.34%)	48.61% (2.44%)	37.48% (3.24%)	0.00% (0.00%)	-00.21% (0.17%)	2.28% (12.87%)	**83.51%** (0.65%)	86.29% (0.11%)

Table S7. Classification: benchmark of classifiers. All performance measures reported as average across 10 folds (in %) are supplemented by the standard deviations in brackets (in %). Bold numbers mark the best performance amongst different classifiers within same category. Panel (A) outlines performance measures of the benchmark algorithms reported in their publications (meaning these results were gathered from different datasets and taxonomies, depending on the specific publication). Panel (B) outlines performance measures of several benchmark algorithms to the proposed "RFSB" classifier methodology. All results were calculated based on the same dataset RepBase+PGSB and the same, proposed taxonomy. The measures are reported for taxonomic levels and overall perspective. In addition, the proposed "RFSB" classifier is applied to TransposonDB and reported in the most right column.

	VC2010		CB4856	
strain	PBSV (pbmm2)	Sniffles (ngmlr)	PBSV (pbmm2)	Sniffles (ngmlr)
SX3383 (QX1791)	665	848	295	289
SX3399 (GXW1)	384	546	278	27
SX3356 (QX1211)	862	831	371	297
SX3351 (DL200)	354	390	260	237
SX3368 (AB1)	262	288	284	233
SX3287 (PS2025)	521	571	257	210
SX3304 (EG4725)	528	417	268	185
SX3306 (ECA36)	927	837	372	299
SX3254 (N2)	13	10	246	212
SX3292 (CB4856)	670	744	2	28
SX3302 (JU394)	208	252	273	234
SX3303 (JT11398)	275	309	262	220
SX3298 (JU775)	494	509	271	55
SX3295 (LKC34)	378	336	261	203
SX3367 (DL238)	671	861	279	287
SX3305 (ECA248)	454	411	271	225
SX3296 (MY23)	555	472	279	187
SX3286 (EG4946)	277	272	270	214
SX3350 (PB306)	458	540	284	258
SX3341 (JU751)	431	424	263	192

Table S8. Detection: Number of observed transposition events. This table shows the number of observed transposition events for different probe reference genome combinations, alignment and structural variant calling tools.

	Number of transposition events									
	Snifles-NGMLR				PBSV-PBMM2					
	Total		TotalBP		Total		TotalBP		Phylogenetic distance	
**strain**	**VC2010**	**CB4856**	**VC2010**	**CB4856**	**VC2010**	**CB4856**	**VC2010**	**CB4856**	**VC2010**	**CB4856**
AB1	288	233	2528558	864269	262	284	450579	907524	0.023819	0.094459
CB4856	744	28	3508318	86028	670	2	1350133	11185	0.096336	0
DL200	390	237	1260330	918529	354	260	737484	613060	0.078775	0.144549
DL238	861	287	5340946	1120948	671	279	1635629	701238	0.100631	0.077201
ECA248	411	225	1674096	828618	454	271	861328	978863		
ECA36	837	299	5547803	933204	927	372	2241605	779615	0.193356	0.185248
EG4725	417	185	1116717	976099	528	268	1077970	542523	0.063796	0.104196
EG4946	272	214	948485	1126127	277	270	839962	797286	0.036709	0.093291
GXW1	546	27	1990299	42255	384	278	784283	624929	0.036727	0.096107
JT11398	309	220	866883	1137900	275	262	557395	664228	0.027652	0.093426
JU394	252	234	1413635	1200411	208	273	747156	795337	0.014571	0.109029
JU751	424	192	4025712	880873	431	263	971156	842471	0.043152	0.099734
JU775	509	55	2324057	198857	494	271	1175322	589865	0.115199	0.165997
LKC34	336	203	935153	763459	378	261	909664	710945	0.037956	0.088754
MY23	472	187	2079515	480242	555	279	1227010	500555	0.08266	0.139242
N2	10	212	39344	576541	13	246	20414	520726	0	0.096336
PB306	540	258	2226567	1042300	458	284	854083	735233	0.056832	0.113414
PS2025	571	210	3381679	856256	521	257	1018228	738095	0.071363	0.127945
QX1211	831	297	3556466	936120	862	371	1895254	723441	0.194891	0.186783
QX1791	848	289	5571466	1232941	665	295	1521737	860000	0.105931	0.097823

Table S9. Detection: Genetic distance and number of transposition events found. This table shows the number of observed transposition event candidates, the length of their mask in bp, and the phylogenetic distance of probe and reference genome.

	**TERL**		**TopDown**		**NLLCPN**		**HC LGA**		**HC GA**		**RFSB**	
**amount**	**av**	**std**	**av**	**std**	**av**	**std**	**av**	**std**	**av**	**std**	**av**	**std**
**10%**	104.86	7.70	28.52	6.19	370.97	10.42	16.67	0.96	22.83	11.56	440.12	66.33
**20%**	169.85	5.56	53.53	3.53	577.79	16.10	32.83	1.08	53.42	15.84	934.80	132.24
**30%**	308.44	92.67	101.86	24.20	698.64	114.03	50.93	3.86	101.39	71.46	1,253.60	116.50
**40%**	398.03	115.80	152.78	20.30	824.14	139.08	75.88	2.07	133.85	121.72	1,355.46	138.52
**50%**	556.83	215.54	231.15	34.42	1,043.13	238.42	109.04	4.44	145.75	217.46	2,135.21	53.22
**60%**	685.97	270.85	287.52	48.11	1,206.68	294.26	140.42	3.44	235.74	265.43	2,911.57	51.22
**70%**	766.33	348.24	337.80	61.92	1,439.53	370.41	176.86	4.11	247.21	313.83	3,383.72	203.48
**80%**	924.98	451.49	423.68	81.78	1,639.20	470.50	206.09	16.16	279.55	405.28	4,351.43	124.92
**90%**	1,083.93	523.47	508.52	86.70	1,845.06	551.04	274.06	6.83	305.89	482.08	4,974.67	103.25
**100%**	1,187.94	602.56	574.38	108.25	1,975.98	620.07	330.81	8.74	332.88	560.98	5,894.92	97.56

Table S10. Classification Runtime Benchmark for Inference: The experiments were conducted using different amounts of the RepBase+PGSB database (reported in %). Inferences were conducted using trained models on whole RepBase+PGSB database. All runtimes reported as average across 10 experiments (in seconds) are supplement by the standard deviations (in seconds).

cture(0,0)(-30,0)10 (-30,-5)(0,1)10 (-35,0)(1,0)30 (0,30)10 (-5,30)(1,0)10 (0,35)(0,-1)30 picturepicture(0,0)(30,0)10 (30,-5)(0,1)10 (35,0)(-1,0)30 (0,30)10

	TERL		TopDown		NLLCPN		HC LGA		HC GA		RFSB	
**amount**	**av**	**std**	**av**	**std**	**av**	**std**	**av**	**std**	**av**	**std**	**av**	**std**
**10%**	76.05	16.52	332.17	47.70	284.25	84.39	958.74	100.68	66.27	10.00	408.56	60.82
**20%**	82.70	14.24	687.22	86.06	393.64	72.24	484.88	76.42	325.39	68.21	848.48	134.79
**30%**	66.08	79.97	840.34	65.10	349.07	287.89	377.30	62.03	817.04	138.04	1,466.01	139.29
**40%**	119.79	110.60	1,138.71	105.39	567.19	385.25	549.90	24.18	1,117.38	267.54	1,234.72	167.88
**50%**	196.65	212.90	1,471.42	75.39	882.72	734.69	766.55	73.54	1,329.86	231.95	1,936.55	208.61
**60%**	256.18	268.45	1,701.08	94.03	1,070.82	865.75	969.57	27.98	1,706.84	303.15	2,624.15	301.07
**70%**	278.47	337.92	2,074.87	152.94	1,220.04	1,112.73	1,220.24	51.59	2,066.18	251.82	3,054.68	336.56
**80%**	381.56	449.34	2,268.66	172.59	1,571.47	1,428.32	1,414.55	126.04	2,226.74	449.72	3,926.72	440.06
**90%**	454.63	521.81	2,589.61	257.26	1,850.36	1,693.01	1,866.12	75.88	2,429.10	524.26	4,482.80	463.53
**100%**	514.49	595.92	3,236.85	626.59	2,052.93	1,937.69	2,231.47	70.49	2,741.35	489.30	5,311.97	541.83

Table S11. Classification Runtime Benchmark for Training: The experiments were conducted using different amounts of the RepBase+PGSB database (reported in %). All runtimes reported as average across 10 experiments (in seconds) are supplement by the standard deviations (in seconds).
